# Extracellular vesicles from highly invasive melanoma subpopulations increase the invasive capacity of less invasive melanoma cells through mir-1246-mediated inhibition of CCNG2

**DOI:** 10.1186/s12964-024-01820-6

**Published:** 2024-09-16

**Authors:** Tim Kingreen, Stefanie Kewitz-Hempel, Christian Rohde, Gerd Hause, Cord Sunderkötter, Dennis Gerloff

**Affiliations:** 1 Department of Dermatology and Venereology, Martin-Luther-University Halle-Wittenberg, Halle (Saale), Germany; 2 Department of Internal Medicine V, Heidelberg University Hospital, Heidelberg, Germany; 3 Biocenter, Martin-Luther-University Halle-Wittenberg, Halle (Saale), Germany

**Keywords:** Extracellular vesicles, miRNAs, Invasion, Melanoma

## Abstract

Invasive growth is a critical process in tumor progression, requiring the activation of various molecular processes in tumor cells at the invasive front. Intercellular communication between heterogeneous tumor cells enhances cellular activation and adaptation to specific microenvironments. One mechanism of intercellular communication is the delivery of miRNAs through tumor cell-derived extracellular vesicles (EVs). In this context we have observed that conditioned media from a highly invasive cell subpopulation (BLM-HI) enhances the invasive capacity of the parental cell line (BLM). Therefore, we hypothesized that this complex change of cellular behavior is influenced by EV-transported miRNAs. The treatment of BLM cells with EVs derived from BLM-HI cells resulted in a significantly enhanced invasive capacity, as observed in Matrigel-embedded spheroids and in 2D Boyden chamber assays, with a dose-dependent effect. Conversely, the invasive capacity of BLM cells was reduced when secretion of EVs was inhibited by a sphingomyelinase inhibitor. To investigate the molecular mechanisms behind this effect, we performed next-generation sequencing and identified an enrichment of miR-1246 in these EVs. In functional analyses we demonstrated that both the EV mediated delivery of miR-1246 as well as overexpression contributes to the enhanced invasiveness of BLM cells. We identified a binding site of miR-1246 in the 3’UTR of cyclin G2 (CCNG2) and demonstrated direct binding by a luciferase reporter assay.

Increased expression of CCNG2 has been associated with cancer metastasis and poor patient outcomes in other malignancies. Our study demonstrates that intercellular communication contributes to the transfer of properties, such as increased invasive capacity, between heterogeneous melanoma cells via EV-transported miRNAs.

**Supplementary Information:**

The online version contains supplementary material available at 10.1186/s12964-024-01820-6.

## Background

Metastasis and invasive growth of solid tumors are fundamental processes of tumor progression that reduce therapeutic options and survival of patients. During invasion and metastasis, tumor cells must induce various molecular processes to spread from their primary tumor to distant sites or organs. In these multistep processes, such as tumor cell dissociation from the primary tumor, invasion, intravasation and extravasation, and ultimately colonization and reproliferation in distant organs, tumor heterogeneity and plasticity are of particular importance [1].

Phenotypic plasticity is crucial, as tumor cells must tolerate and adapt to various stressors and changing environments. Intratumoral heterogeneity is determined by the tumor microenvironment, which exerts selective pressure on tumor cells. These complex mechanisms lead to distinct evolutionary intratumoral subpopulations that may differ in their genetics, epigenetics, transcriptome, and proteome, resulting in varying functional properties such as high invasive capacities or a highly proliferative state [2].

Although metastasis is often understood as a single-cell process, clusters consisting of different subpopulations of tumor cells with distinct invasive protential have been described at tumor-invasive fronts, in the bloodstream, and colonizing distant organs [3, 4]. This suggests that cooperation between tumor cells from different tumor subpopulations is critical for metastasis. Therefore, we hypothesized that cooperation of different cell clones is also important for the first processes of metastasis, i.e.: (1) dissociation of tumor cells from the tumor composite as well as (2) invasion of these cells into the tissue. One mechanism for this cooperation is the exchange of genetic information and functional molecules by means of extracellular vesicles (EVs). Although, intercellular communication was once primarily considered to be mediated by cytokines, EVs and their contents are increasingly being recognized as additional powerful mediators, partially because they also convey nucleic acids. They are nanoparticles surrounded by a lipid membrane that are formed and taken up by all cells. They transport a package of functional molecules such as proteins, DNA, RNAs and non-coding RNAs (e.g. miRNAs), which after uptake, affect the phenotype and function of recipient cells [5, 6]. Depending on their biogenesis, size, loading and function, they are mainly divided into the three classes: exosomes (approximately 50–200 nm), microvesicles (200–1000 nm) and apoptotic bodies (> 1000 nm).

For various cancer types, tumor cells have been demonstrated to affect miscellaneous non-tumor cells as well as tumor cells in the microenvironment by releasing EVs. We have revealed that melanoma cell-derived EVs contribute to tumor-associated macrophage (TAM) differentiation [7]. Other studies showed that EV-mediated manipulation of cells in the microenvironment, is leading to the induction of tumor-promoting macrophages [8], regulatory T cells [9] or even to the inhibition of activated effector T cells and thus to immune evasion [10, 11]. In addition, stromal cells such as fibroblasts are reprogrammed to become carcinoma associated fibroblasts (CAFs) by the uptake of tumor cell-secreted EVs, which, among other effects, support tumor growth and metastasis [12]. Another report revealed that EVs derived from melanoma cells mediate the function of tumor endothelial cells [13]. In the context of collaborative activity among distinct subpopulations of tumor cells within tumors, studies have confirmed that extracellular vesicles (EVs) impart therapy resistance, promote proliferation, and enable invasion or metastasis. For melanoma, EVs have been demonstrated to contribute to inducing a pro-invasive phenotype, amongst other effects [14, 15]. Delivery of the microRNAs (miRNAs) let-7a, let7i, and miR-191 into melanocytes results in the repression of E-cadherin and the upregulation of SNAIL2, VIM, and ZEB2, respectively [15]. The latter study confirmed the functional relevance of miRNAs that are transported by EVs derived from melanoma cells. In this context we demonstrated that EVs containing miR-125b-5p from melanoma cells induced a pro-inflammatory and pro-angiogenic subtype of macrophages. This effect was mediated by suppressing the expression of LIPA [7].

miRNAs are small, non-coding RNAs (≈ 22 nucleotides) and are among the functional molecules transported from one cell to another by EVs. In the cytoplasm miRNAs are bound by the RNA induced silencing complex (RISC), directing it to a complementary binding site in the 3’ untranslated region (3’UTR) of a target RNA. Binding of the miRNA-RISC complex to this mRNA binding site leads to inhibition of protein translation [16, 17]. Since miRNA-mRNA binding is only partially complementary, one miRNA can target many potential mRNAs. Furthermore, mRNAs often have multiple potential binding sites of different miRNAs in their 3’UTR, thus multiple miRNAs can regulate protein expression of a gene and act synergistically. Therefore, their dysregulation or their EV-mediated accumulation has a major impact on tumor progression [18–21] or on the development of resistance to certain therapies [22, 24].

The focus of our research is on the functionality of miRNAs in EVs derived from malignant melanoma [7, 24]. Our previous analysis of miRNAs in various melanoma models has revealed quantitative differences [24]. Additionally, we demonstrated that miRNAs can function as either oncomiRs [7] or tumor suppressive miRNAs [23] in malignant melanoma.

Since we have observed that the miRNA cargo is different in EVs derived from various melanoma cell lines or from normal melanocytes [24], we wondered if miRNAs derived from highly invasive tumor cells would mediate increase the invasive capacity of less invasive tumor cells.

Our investigations revealed an increased accumulation of miR-1246 in EVs derived from highly invasive melanoma cell subpopulation (BLM-HI). The delivery of this miR-1246 enhances the invasive capacity of the parental melanoma cell line (BLM) by downregulation of CCNG2.

## Methods

### Cell cultures

Melanoma cell lines BLM, BLM-HI, WM35, WM9, WM902B and A375 were cultured in DMEM (Gibco, Thermo Fisher Scientific, Waltham, Massachusetts, USA) supplemented with 10% fetal calve serum (FCS) (Sigma Aldrich, Taufkirchen, Germany) and 1% penicillin-streptomycin (Sigma Aldrich, Taufkirchen, Germany). Melanoma cell lines were provided by the Department of Dermatology, University of Münster, Germany. The principal cell line under examination in this study is a human cell line, designated BLM. It was derived from the BRO cell line through a selection assay and is derived from a lung metastasis. Primary normal human epidermal melanocytes (NHEM) were isolated in our laboratory from juvenile foreskins and cultured in medium 254 (Thermo Fisher Scientific, Waltham, Massachusetts, USA) including human melanocyte growth supplement (HMGS) and 1% penicillin-streptomycin. All cell lines were incubated at 37 °C and 5% CO_2_.

### Isolation and analysis of small extracellular vesicles

EVs were isolated and characterized according to the 2018 consensus statement on minimal information for studies of extracellular vesicles (MISEV2018) [25]. Cells were cultured for 48–72 h in DMEM supplemented with 10% exosome depleted FCS (Thermo Fisher Scientific, Waltham, Massachusetts, USA) and 1% penicillin-streptomycin. Supernatants (30 ml) were collected and centrifuged for 10 min at 300 g to remove cells and cell debris, followed by 30 min at 10,000 g to remove larger vesicles. Afterwards the supernatants were filtered through a 0.2 μm filter and centrifuged at 100,000 g for 2 h. Centrifugation was performed using a Sorvall WX + Ultra Centrifuge, with SureSpin 632 rotor (k-factor 194) (Thermo Fisher Scientific, Waltham, Massachusetts, USA). EVs were resuspended in PBS. In addition EVs were enriched using size exclusion chromatography (sec) according manufacturer’s instructions (Cell Guidance Systems, Cambridge, UK). EV analysis was performed by nanoparticle tracking analysis (NTA) using a NanoSight NS300 (Malvern Panalytical, Kassel, Germany). Therefore, EVs were isolated and analysed from three independent biological samples. Measurements were performed at a controlled temperature of 22 °C. For each sample, three measurements of 30 s were performed. EV concentration and size was calculated by the NanoSight software.

### EV uptake

To confirm the cellular uptake of EVs, isolated exosomes were stained with SYTO^®^ RNASelect™ (Thermo Fisher Scientific) or Bodipy (Thermo Fisher Scientific) according to the manufacturer’s instructions. BLM cells were treated with 10 µg/ml stained EVs for 24 h and analyzed by fluorescence microscopy.

### Transmission electron microscopy (TEM)

To prepare TEM-samples 3 µl of the dispersion were spread onto Cu-grids coated with a formvarfilm. After 1 min of adsorption, excess liquid was blotted off with filter paper. Subsequently the grids were air-dried for 15 s, washed with water (3 times for 1 min), placed on a droplet of 2% aqueous uranyl acetate and drained off after 1 min. The dried specimens were examined with an EM 900 transmission electron microscope (Carl Zeiss Microscopy, Jena, Germany) at an acceleration voltage of 80 kV. Electron micrographs were taken with a Variospeed SSCCD camera SM-1k-120 (TRS, Moorenweis, Germany).

### Immunoblot analyses

Cells and EVs were lysed by RIPA buffer for 30 min at 4 °C. 20 µg of protein extracts were resolved by SDS–PAGE and blotted to nitrocellulose membranes and probed with the following antibodies: anti-CD81 (5A6) (1:200); anti-CD63 (MX-49.129.5) (1:500); anti-ALIX (1A4) (1:250); anti-CANX (AF18) (1:500); anti-HSP70 (3A3) (1:500); anti-GAPDH (0411) (1:1000); anti-VIM (V9) (1:1000); anti-MMP2 (8B4) (1:1000); anti-CCNG2 (1F9-C11) (1:1000), anti-CDH1(1.B.54) (1:1000); anti-CDH2 (13A9) (1:1000) (all Santa Cruz, Dallas, USA) and anti-CD9 (CGS12A) (1:1000) (Cell Guidance Systems, Cambridge, UK). Antibody incubation was performed in 5% milk at 4 °C over night. For antibody detection, blots were incubated for 1 h at room temperature with m-IgGκ BP-HRP (1:5000) (Santa Cruz, Dallas, USA) or anti-mouse IgG-HRP (1:2000) (Cell Signaling Technology, Leiden, Netherlands). Chemiluminescent detection was performed using Amersham ECL Prime (GE Healthcare, Amersham, UK).

### RNA isolation and analyses

Total RNA was extracted from cells or extracellular vesicles using TriFast™ reagent (Peqlab, Erlangen, Germany), according manufacturer’s protocol. RNA quality and quantity was analysed by Agilent bioanalyser (Agilent, Santa Clara, California, USA). MiRNA quantification was performed by qRT-PCR using TaqMan^®^ MicroRNA Reverse Transcription Kit and TaqMan^®^ Universal Master Mix II according manufacturer’s instructions (Thermo Fisher Scientic). Values were normalized by RNUB6 for cells, while for extracellular vesicles values were normalized to synthetic spike in cel-miR-39. Relative fold changes were calculated by 2^-ΔΔCt^ method [26], comparing the values to the mean of the control group. MiRNA assays were purchased from Thermo Fisher Scientific (Thermo Fisher Scientific, Waltham, Massachusetts, USA).

### RNase protection assay

BLM-HI-derived EVs were treated with RNase (0.4 mg/ml) alone or in combination with Triton X-100 (0.1%) at 37 °C for 30 min. RNase was inhibited by incubation at 70 °C for 10 min followed by RNA isolation. The quantity of miR-1246 was determined through qRT-PCR. The values were normalized to cel-miR-39 spike-in.

### Treatment of BLM and BLM HI cells

To block miR-1246 function or to overexpress miR-1246 cells were transfected with 200 nM LNA-miR-1246, 200 nM LNA control (Power Inhibitor), 200 nM miR-1246 mimic or 200 nM mimic negative control by lipofectamine 3000 (Thermo Fisher Scientific, Waltham, Massachusetts, USA) in 2D cell culture. After 24 h in cell culture cells were used to generate spheroids or for further analyses. LNA-miR-1246, LNA control, miR-1246 mimic and mimic negative control were purchased by Qiagen (Hilden, Germany). To inhibit secretion of EVs, spheroids were treated for 48 h with 15 µM GW-4896 (Merck, Darmstadt, Germany) or DMSO as control.

### Next generation sequencing

mRNA-sequencing was performed using 2 µg of RNA for each sample: library prep and sequencing was performed by Genewiz (Leipzig, Germany). Library prep was based on poly-A-tail selection; sequencing was performed on an Illumina NovaSeq platform resulting in ~ 20 million reads per sample. Raw data was quality checked (80% bases Q ≥ 30) and trimmed via Trim Galore! v0.4.3.1. The Reads were mapped via RNA STAR v2.6.0b-2 to human genome hg38. The differential gene expression analysis was performed according to edgeR v 3.24.1 usage.

For miRNA sequencing 10 ng of total RNA was used in the small RNA protocol with the NEXTflex Small RNA-seq Kit v3 (Bioo Scientific) according to the instructions of the manufacturer. A pool of libraries was used for sequencing at a concentration of 10 nM. Sequencing of 1 × 75 bp was performed with an Illumina NextSeq 550 sequencer at the sequencing core facility of the IZKF Leipzig (Faculty of Medicine, University Leipzig) according to the instructions of the manufacturer. Demultiplexing of raw reads, adapter trimming and quality filtering was done according to Stokowy et al. [27], using the adapter sequences of the NEXTflex kit containing random bases next to the library insert. Mapping against the human reference genome (hg38) and miRbase reference sequences (v22) was done using Bowtie2 [28]. Read counts were calculated with the R bioconductor package Rsamtools (http://bioconductor.org/packages/release/bioc/html/Rsamtools.html) and normalised using the DESeq2 [29] and EdgeR [30] R bioconductor packages.

### Invasion assays

Invasion assay was perfomed using Matrigel (Corning, Sigma-Aldrich, Taufkirchen, Germany) coated transwell filters (8 μm pores, Sarstedt, Nümbrecht, Germany) placed in 12-well plates following the manufacturer’s protocol. To measure invasion 2 × 10^5^ cells were seeded in DMEM medium without FCS onto the Matrigel-applied transwell filter. As chemoattractant, DMEM containing 10% FCS, was added to the bottom of the well. After 24 h, matrigel and non-invading cells were removed with a cotton swab. Cells invaded the membran were fixed with 100% methanol and stained using gentian violet. To count cell numbers, five separate areas per chamber were photographed using a Keyence BZ-X microscope.

### Spheroide assay

For the formation of 3D spheroids 5000 cells were seeded into a 96 well plate with U bottom (Greiner Bio-One, Kremsmünster, Austria) using 100 µl and subsequently centrifuged at 300 g for 5 min. After three days 50 µl matrigel was added. The spheroids were photographed every 24 h and their invasive area was calculated using QuPath 0.4.3. software [31].

### Luciferase reporter assay

To confirm miR-1246 binding to CCNG2 3′UTR we used luciferase reporter clone for human CCNG2 3′UTR (HmiT149050-MT06, Genecopoeia (Rockville, Maryland, USA)) and luciferase control reporter construct (CmiT000001-MT06, Genecopoeia (Rockville, Maryland, USA)). Constructs (1 µg/ml) were co-transfected with miR-1246 mimic (200 nM) or with ctrl-mimic (200 nM) (Qiagen, Hilden, Germany) in 293T cells using Lipofectamine 3000 according the manufacturer’s instructions. The relative luciferase activity was measured 24 h after transfection using Luc-Pair Duo-Luciferase Assay Kits 2.0 (Genecopoeia (Rockville, Maryland, USA)) following the manufacturer’s instructions.

### Gene Set enrichment

Gene set enrichment analyses (GSEA) were performed using GSEA 4.0.3. software.

### Heatmaps and statistics

For the statistical analyses and graphical representation, Qlucore Omics Explorer and Graph Pad Prism software was used. Differential expression fold-changes and p-values of the NGS data were calculated by Qlucore Omics Explorer. To prove the statistical significands of the data, two tailed Student’s t-test or Mann-Whitney U-test was performed, depending on Gaussian distribution, which was evaluated by the Levene test. A p-value ≤ 0.05 was considered as statistical significant. For comparison of multiple groups or conditions One way ANOVA analyses followed by by Dunnett’s comparisons test against the control group were performed. All graphs represent the results of at least 3 independent experiments.

## Results

### BLM-derived sub cell line BLM-HI shows increased invasion ability

**Fig. 1:**
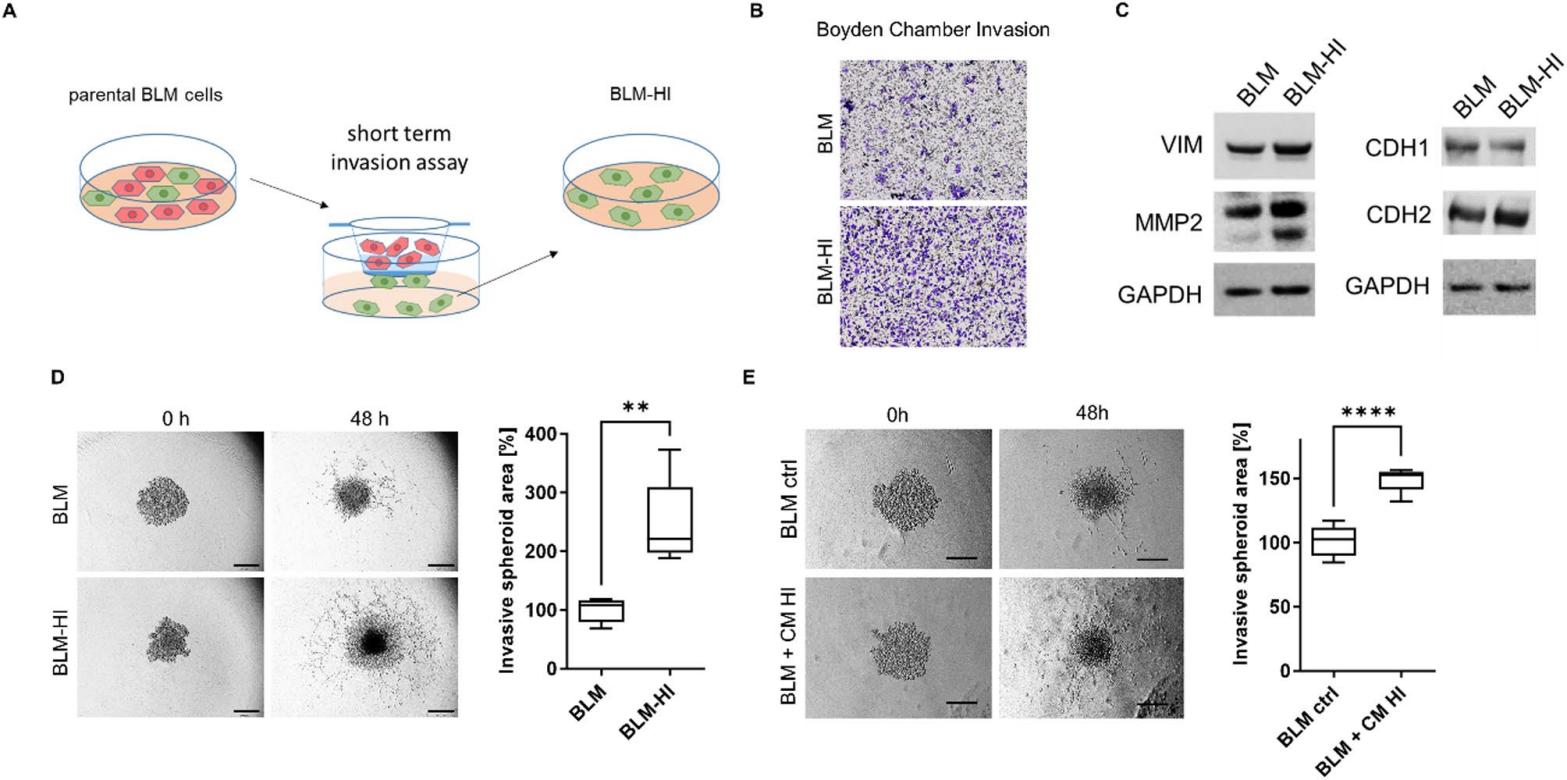
BLM-derived sub cell line BLM-HI shows increased invasion ability. (**A**) Scheme represents the separation of BLM-HI subpopulation. (**B**) Exemplary pictures show invasion capacity of BLM and BLM-HI cells in 2D invasion assays (matrigel coated Boyden chamber). (**C**) Western blot analyses for VIM, CDH1 (E-Cadherin), CDH2 (N-Cadherin) and GAPDH of BLM and BLM-HI cells. 3D invasion assay of matrigel embedded spheroids of (**D**) BLM and BLM-HI cells or (**E**) BLM cells treated with conditioned medium of BLM-HI cells (CM HI) or without (ctrl). Graphs show the measured invasive area of at least 5 independent spheroids (**p < 0.01, ****p < 0.0001)

Given that melanoma cell lines are a heterogeneous mixture of cell sub-populations, we wanted to investigate whether invasive sub-populations can transfer their ability to other less invasive melanoma cells. To accomplish this, we carried out invasion assays using Boyden chambers to isolate highly invasive BLM cells (BLM-HI) from their parental counterpart BLM (Fig. 1A). After incubation for 8 h, we collected rapidly invasive cells from the bottom of the Boyden chamber and cultured them under the same conditions as the parental BLM cells for expansion. Comparative invasive capability analysis of BLM and BLM-HI cells was carried out using both 2D Boyden chamber system and 3D matrigel-embedded spheroids. We observed a significant increase in invasion ability of BLM-HI cells in both assays (Fig. 1B/D). In Western blot, BLM-HI cells showed increased expression of vimentin (VIM), N-cadherin (CDH2), and higher levels of active truncated form of metalloproteinase 2 (MMP2), while E-cadherin (CDH1) was decreased, all compared to parental BLM cells (Fig. 1C).

Since tumor cells have the ability to influence their environment through intercellular communication, we investigated whether the highly invasive BLM-HI cells could enhance the invasive potential of the parental BLM cells. We analysed the invasion of matrigel-embedded spheroids from BLM cells both with and without the addition of BLM-HI conditioned medium (CM HI). The addition of CM HI significantly enhanced the invasion of BLM cells (Fig. 1E). Treatment of BLM spheroids with conditioned media derived from BLM cells, did not increase the invasive capacity (Figure S1).

Thus, our results show that the increased invasive ability of BLM-HI cells can be transferred to the parental BLM cell line via BLM-HI conditioned medium.

### Extracellular vesicles derived from BLM and BLM-HI cells show comparable characteristics

**Fig. 2:**
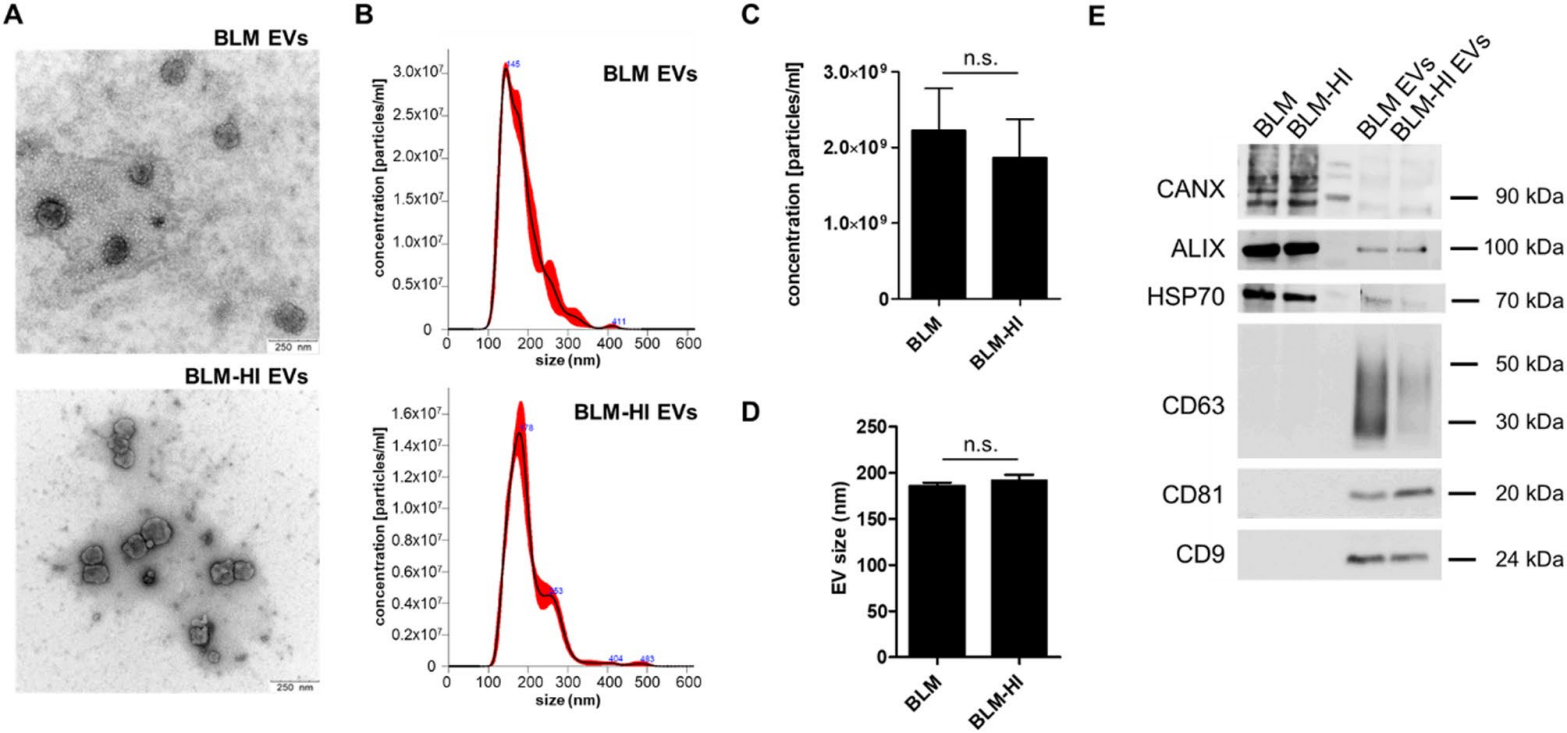
Extracellular vesicles derived from BLM and BLM-HI cells show comparable characteristics. Analyses of EVs by (**A**) transmission electron microscopy (TEM) (scale bar represents 250 nm) and (**B**) Nanoparticle tracking analysis (NTA). Bars represent (**C**) size distribution and (**D**) concentration of EVs isolated from BLM or BLM-HI cells. (**E**) Western Blot analyses of EV surface markers CD9, CD63 and CD81 (tetraspanins) and of cytosolic proteins ALIX and HSP70. Calnexin serves to detect cellular contaminations. Bars represent the mean ± standard deviation of at least 3 independent measurements. (n.s. = not significant)

To determine whether EVs are involved in enhancement of BLM cell invasion, we characterized and compared the EVs of BLM and BLM-HI cells. We isolated EVs using differential centrifugation, including ultracentrifugation and size exclusion chromatography (SEC), as previously described. Transmission electron microscopy (TEM) showed that EVs from BLM and BLM-HI cells had a uniform round structure and similar size distribution (Fig. 2A).

Nanoparticle tracking analysis (NTA) confirmed the EVs comparable size distribution (Fig. 2B/C) and concentration (Fig. 2D). In Western blot studies, EVs from both cell lines exhibited similar expression of the assigned EV markers, such as tetraspanins CD9, CD63, and CD81, and the cytosolic proteins HSP70 and ALIX (Fig. 2E). Uptake assays were performed to investigate the functionality of the isolated EVs. For this purpose, the isolated EVs were stained with Syto Green (RNA staining) or Bodipy (ceramide staining) and added to the BLM cells. The uptake of isolated EVs by BLM cells was comparable whether they were derived from BLM cells or BLM-HI cells (Figure S2).

The EVs obtained from both BLM and BLM-HI cells display similar characteristics, including size distribution, concentration, surface marker expression and uptake.

### EVs derived from BLM-HI cells enhance the invasive capability of parental BLM cells

**Fig. 3:**
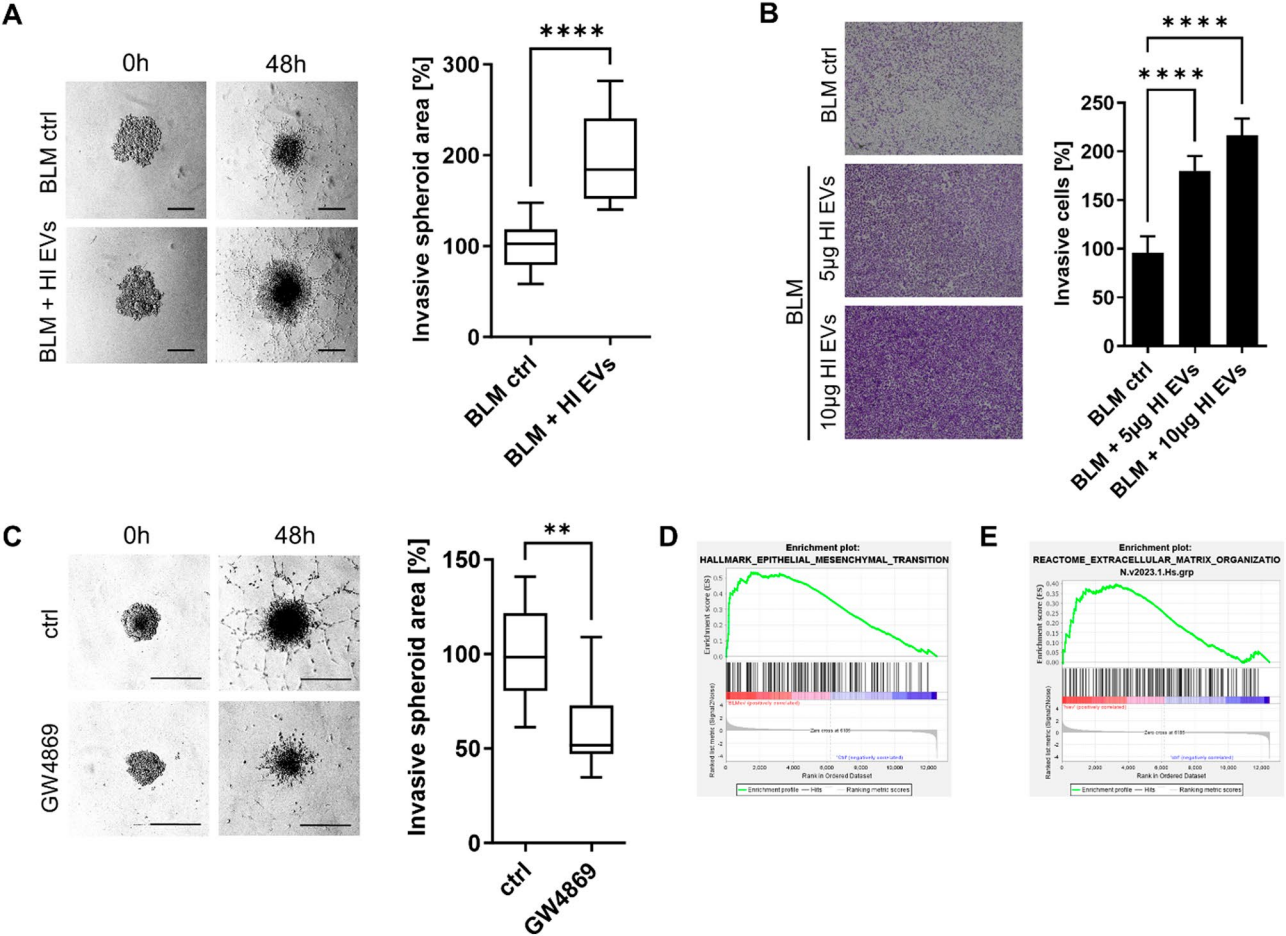
EVs derived from BLM-HI cells enhance the invasive capability of parental BLM cells. Invasion capacity of BLM cells treated with or without EVs derived from BLM-HI cells was analysed in (**A**) matrigel embedded spheroids or (**B**) Boyden chambers. Bars represent the mean ± standard deviation of at least 3 independent experiments. (**C**) 3D invasion assay of BLM cells treated with and without SMase inhibitor (GW4869) to block secretion of EVs. Boxplots represents the measurements of the invasive area of at least 5 spheroids. (**p < 0.01; ****p < 0.0001). (**D**) Heatmap shows differential gene expression of BLM cells with and without addition of EVs derived from BLM-HI cells. (**E**) Gene set enrichment analyses (GSEA) of BLM cells treated with and without EVs derived from BLM-HI cells show an enrichment of genes associated with epithelial mesenchymal transition (EMT) and with extracellular matrix organization

BLM spheroids were treated with EVs derived from BLM-HI cells or PBS as a control and embedded in matrigel. BLM spheroids treated with EVs derived from highly invasive BLM-HI cells showed a significantly increased invasive area compared to the control spheroids (Fig. 3A). EVs derived from BLM cells did not enhance the invasion of BLM spheroids, which was similar to treatment with BLM-conditioned media. (Figure S1). An enhanced invasion of BLM cells was also observed in 2D Boyden chamber invasion assays when treated with EVs derived from BLM-HI cells (Fig. 3B). The invasion capability of BLM cells was enhanced by BLM-HI cell derived EVs in a dose-dependent manner (Fig. 3B). To confirm that increased BLM cell invasion was mediated by EVs, we performed 3D spheroid invasion assays by treating spheroids with GW4896, a sphingomyelinase (SMase) inhibitor, to inhibit EV secretion and autologous stimulation of the cells, or DMSO as a control. We observed a significant reduction in BLM cell invasion into matrigel after EV secretion was blocked with GW4896 (Fig. 3C).

To identify the molecular changes between BLM cells after treatment with or without BLM-HI EVs, next generation sequencing (NGS) analyses were performed (Fig. 3D). NGS analysis revealed differential gene expression in BLM cells after treatment with EVs derived from BLM-HI cells. In Gene set enrichment (GSEA) analyses we found an increased expression of genes related to epithelial-mesenchymal transition (EMT) and extracellular matrix organization after treatment with BLM-HI EVs (Fig. 3E).

In conclusion, our observations of EV mediated increased invasiveness are supported by the results of the NGS analyses.

### miR-1246 is enriched in EVs derived from BLM-HI cells

**Fig. 4:**
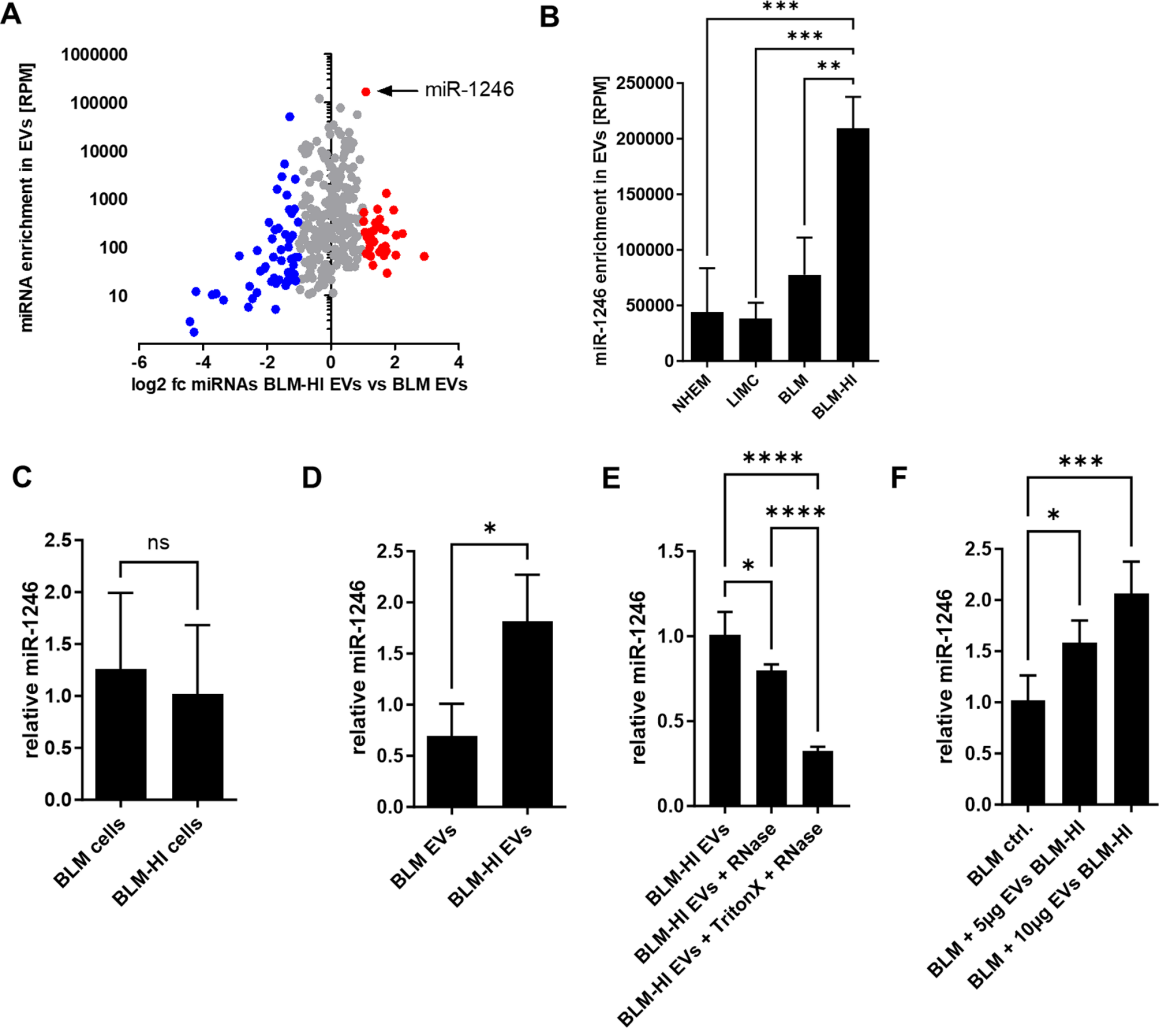
miR-1246 is enriched in EVs derived from BLM-HI cells. (**A**) Volcano plot shows differential enriched miRNAs in EVs derived from BLM or BLM-HI cells. (**B**) NGS data show accumulation of miR-1246 in EVs derived from normal melanocytes (NHEM), low invasive melanoma cells (LIMC (WM35, WM902B, WM9, A375)), BLM and BLM-HI. (**C**) Expression of miR-1246 was analysed in BLM and BLM-HI cells by qRT-PCR. (**D**) qRT-PCR analysis shows accumulation of miR-1246 in EVs derived from BLM or BLM-HI cells. (**E**) RNase protection assay: qRT-PCR analysis of miR-1246 in EVs after indicated treatment. (**F**) qRT-PCR revealed the enrichment of miR-1246 in BLM cells after treatment with mentioned amount of EVs derived from BLM-HI cells. Bars represent the mean ± standard deviation of at least 3 independent experiments (n.s. not significant; *p < 0.05; ***p < 0.001; **** *p* < 0.0001)

Our previous studies revealed that EVs transport various microRNA (miRNA) cargo depending on their cell of origin, with the potential to modulate the characteristics of recipient cells. Consequently, we conducted NGS analyses of small RNAs obtained from EVs originating from BLM cells and BLM-HI cells. We observed a differential distribution of 89 miRNAs, with 55 being present in higher levels in EVs derived from BLM cells and 34 being more enriched in EVs derived from BLM-HI cells (Fig. 4A). miR-1246 was identified as the most abundant miRNA in EVs and was also found to be the most enriched in EVs from BLM-HI cells compared to BLM-secreted EVs. Compared to EVs derived from primary normal human epidermal melanocytes (NHEM), melanoma cell lines with lower invasive potential (LIMC (WM35, WM902B, WM9, A375)) and parental BLM cells, EVs derived from BLM-HI cells showed the highest enrichment of miR-1246 (Fig. 4B). Further analysis by qRT-PCR showed no difference in miR-1246 expression in BLM and BLM-HI cells (Fig. 4C), but it supported the results of our NGS analysis detecting an increased accumulation of miR-1246 in BLM-HI EVs compared to EVs derived from parental BLM cells (Fig. 4D). To verify that miR-1246 is encapsulated in the EVs, we performed a RNase protection assay. miR-1246 was protected from RNase degradation in the EVs, whereas Triton X disruption of the EVs resulted in RNase degradation of miR-1246 (Fig. 4E). Since we found an increased amount of miR-1246 in EVs derived from BLM-HI cells we wanted to verify that it is indeed delivered into BLM cells. Therefore, we treated BLM cells without or with different amounts of BLM-HI EVs. By qRT-PCR we observed an enrichment of miR-1246 after treatment with EVs, which was further increased by higher amounts of EVs (Fig. 4F). In contrast, treatment with EVs derived from BLM cells did not result in an additional increase in miR-1246 accumulation in BLM cells. (Figure S3).

Our results revealed differences in the miRNA-load between EVs derived from parental BLM cells and BLM-HI cells and as well as a delivery of miR-1246 via these EVs.

### miR-1246 participates to enhanced invasiveness of BLM cells

**Fig. 5:**
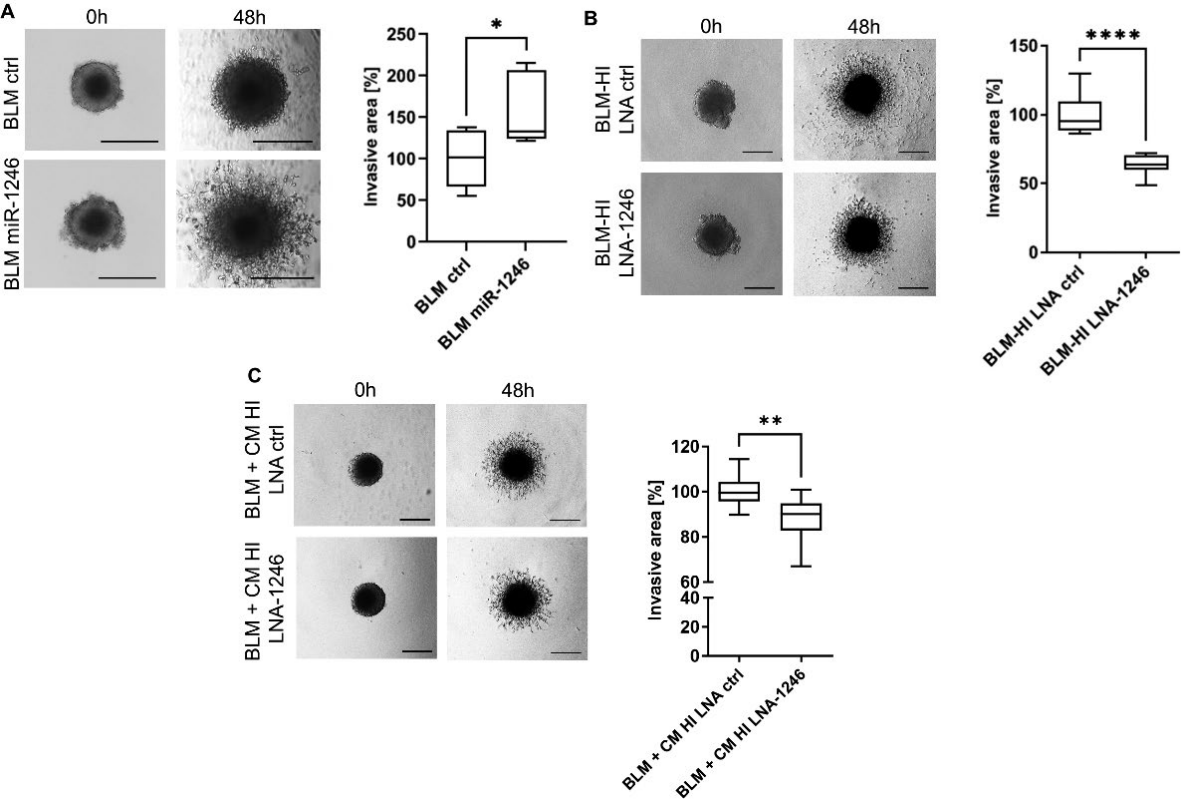
miR-1246 participates to enhanced invasiveness of BLM cells. 3D invasion assay with matrigel embedded spheroids (**A**) of BLM cells with and without miR-1246 overexpression, (**B**) BLM-HI cells with and without miR-1246 inhibition by locked nucleic acids (LNAs) and (**C**) BLM cells treated with conditioned medium (CM) from BLM-HI cells with and without LNA mediated inhibition of miR-1246. Boxplots represents the measurements of the invasive area of at least 5 spheroids (*p < 0.05; **p < 0.01; ****p < 0.0001)

Since we have demonstrated that miR-1246, which is highly enriched in BLM-HI EVs, is transported into BLM cells, we wanted to examine if it contributes to the enhanced invasion of BLM cells mediated by EVs derived from BLM-HI. Overexpression of miR-1246 by mimics in BLM cells enhances the invasion capacity in 3D spheroid assays (Fig. 5A). Consistent with this result, the block of miR-1246 by specific locked nucleic acids (LNAs) in BLM-HI cells (Fig. 5B) and in BLM cells during treatment with conditioned medium from BLM-HI cells (Fig. 5C) lead to decreased invasive areas of spheroids in 3D invasion assays. These results give evidence that miR-1246 indeed influences the invasive capacity of recipient BLM cells.

### miR-1246 targets CCNG2, which is associated with metastasis and worse patient outcome

**Fig. 6:**
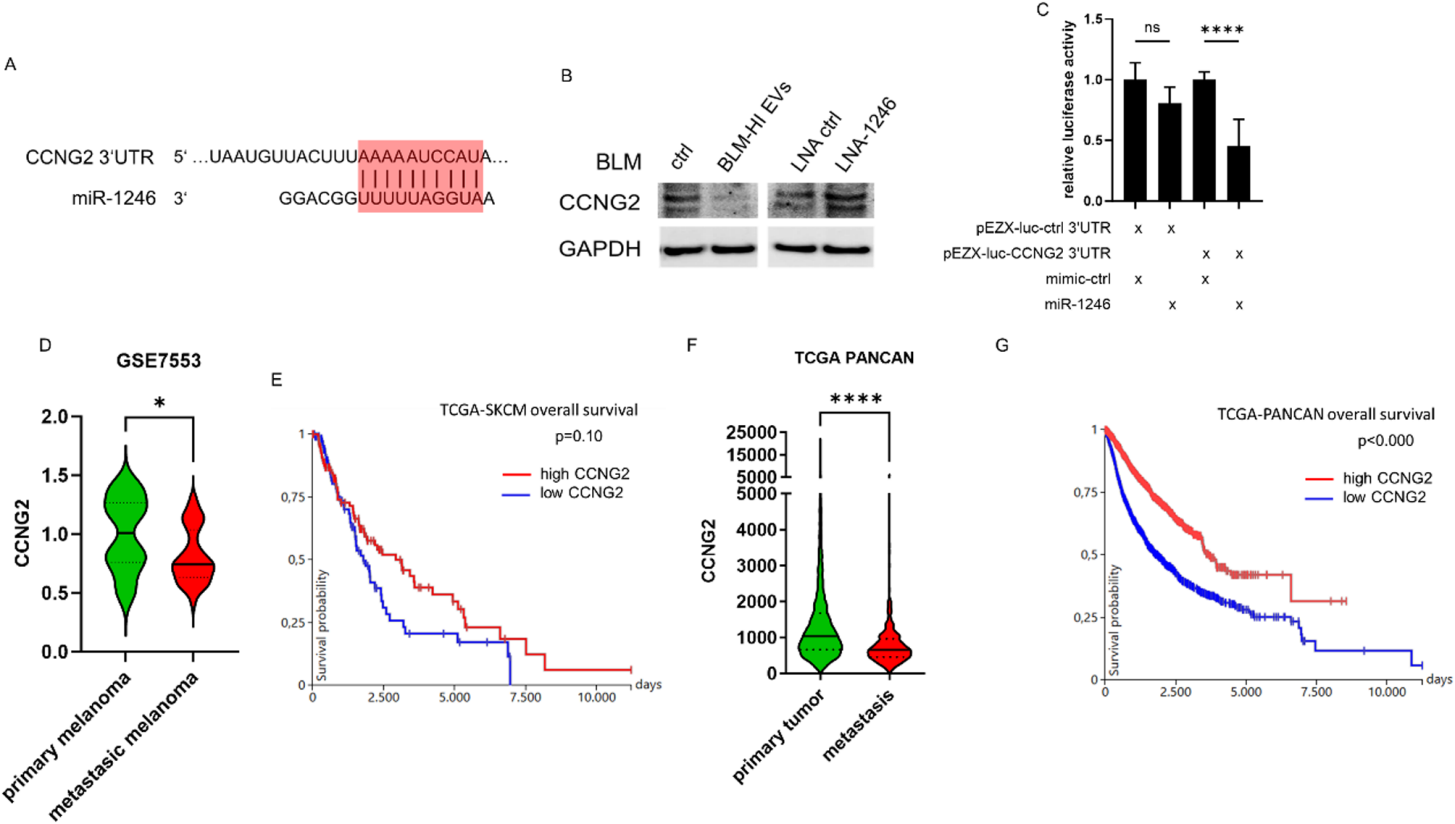
miR-1246 targets CCNG2, which is associated with metastasis and worse patient outcome. (**A**) Target prediction of miR-1246. Targetscan identified a putative seed in the 3’UTR of the CCNG2 mRNA. (**B**) Western Blot analyses of CCNG2 expression in BLM cells after treatment with or without EVs derived from BLM-HI cells and after transfection with locked nucleic acids (LNAs) to block miR-1246. (**C**) Luciferase assay 24 h after co-transfection of pEZX-luc-ctrl 3’UTR (control) or pEZX-luc-CCNG2 3’UTR (CCNG2 3’UTR) with mimic-ctrl (control) or miR-1246 (miR-1246-mimic) in 293T cells. Bars represent the mean ± standard deviation of at least 3 independent experiments (**** *p* ≤ 0.0001; ns: not significant). (**D**) Comparison of CCNG2 expression between primary and metastatic melanoma (public dataset GSE7553). (**E**) Kaplan-Meier curve shows overall survival of metastatic melanoma patients (TCGA-SKCM cohort) dependent on CCNG2 expression. (**F**) Comparison of CCNG2 expression between primary and metastatic tumors (TCGA-PANCAN cohort). (**G**) Kaplan-Meier curve shows overall survival of various cancer entities (TCGA-PANCAN cohort) dependent on CCNG2 expression

To understand how miR-1246 influences invasion of BLM cells, we performed in silico analyses to identify putative targets. Thereby we found a potential binding side for miR-1246 in the 3’UTR of cyclin G2 (CCNG2) (Fig. 6A)When we treated BLM cells with EVs derived from BLM-HI cells the protein level of CCNG2 decreased, while LNA-mediated block of miR-1246 resulted in an increase of CCNG2 protein (Fig. 6B). To demonstrate the direct binding of miR-1246 to the 3’UTR of CCNG2, we performed a luciferase reporter assay. The result shows a decrease in luciferase activity after co-transfection of the CCNG2 3’UTR luciferase construct and miR-1246 mimic, which demonstrates the direct binding of miR-1246 to the CCNG2 3’UTR (Fig. 6C). In addition, in a public data set (GSE7553) we found that CCNG2 expression is decreased in melanoma metastasis compared to primary melanoma (Fig. 6D). Furthermore, data from the TCGA database revealed that low expression of CCNG2 is associated with worse patient overall survival (Fig. 6E). According to our analysis of these results are also true for other cancer entities. In the TCGA pan cancer dataset, including 33 tumor entities, CCNG2 expression is decreased in metastasis compared to primary tumors (Fig. 6F) and low expression of CCNG2 is also associated with worse patient overall survival (Fig. 6G) and decreased progression free survival (Figure S4). Taken together, these results provide evidence for a correlation between high miR-1246 levels and the reduction of CCNG2, which explains higher invasiveness of cancer cells leading to metastasis and poorer patient outcomes.

## Discussion

The objective of our study was to investigate whether miRNAs in EV from highly invasive tumor cells would increase the invasive capacity of less invasive tumor cells. We found that highly invasive cell subpopulation (BLM-HI) enhance the invasive capacity of their parental cell line (BLM) from which they are derived, that EVs derived from BLM-HI cells enhance the invasive capability of parental BLM cells, that miR-1246 was enriched in these EVs derived from BLM-HI cells and that it mediated enhanced invasiveness of BLM cells. It targets CCNG2, which is known to be associated with metastasis and worse patient outcome.

So far, it was known that EVs derived from melanoma cells remodel the behavior and function of tumor cells [14]. Such effects of melanoma cell-derived EVs were described in a previous study showing that knockout of RAB27A, an important mediator of EV cargo composition, in B16-F10 murine metastatic melanoma cells reduced melanoma cell motility in vitro and spontaneous metastasis in vivo [14]. The changes in RAB27A-KO cell behavior were demonstrated to be EV-dependent, as it could be reversed by the exposure to EVs derived from cells re-expressing RAB27A [14], but it was not related with miRNAs.

Increasing evidence suggests that miRNAs carried by extracellular vesicles have crucial functions in regulating the phenotype of recipient cells. In this study, we discovered that mir-1246 is enriched in EVs derived from the highly invasive BLM-HI cell subpopulation and that delivering or overexpressing.

miR-1246 enhances the invasive capabilities of the parental cell line BLM, while its inhibition reduces invasion of BLM-HI cells. Our results are consistent with other studies that have investigated the influence of EV-delivered miRNAs on melanoma cell characteristics. The delivery of miR-222 by EVs into melanoma cells activates the PI3K-AKT pathway, promoting tumor progression by increased proliferation as well as enhanced migration and invasion [32]. Similar results have been shown for miR-106b-5p [33] and miR-494 [34], which are transported by melanoma derived EVs and both enhance cell motility in vitro and induce melanoma lung metastasis in a murine in vivo model. In contrast to our study, these studies identified the investigated miRNAs by comparing benign cells (melanocytes) with melanoma or primary and metastatic tumor stages, whereas we compared EVs from a highly invasive cell subpopulation (BLM-HI) with EVs derived from the parental, less invasive cell line BLM. A study with a comparable focus to ours demonstrated an enrichment of miR-411-5p in EVs of the highly metastatic melanoma cell line M14-POL, which enhanced the metastatic potential of the less metastatic M14-OL cells by their EV-mediated transfer [35]. Although our data showed an enrichment of miR-411-5p in BLM-HI derived EVs, we focused on miR-1246, because it was present in substantially higher levels in EVs from our highly invasive cells than miR-411-5p. These variations in miRNA amounts could be due to differences in the respective melanoma cell lines used in these studies as EV source. Our results, together with the studies mentioned above, thus have elaborated that EV-mediated miRNA transport can enhance invasiveness and metastasis.

We confirmed experimentally that enrichment or overexpression of miR-1246 in BLM cells enhances their invasive capacity, while its block reduces invasiveness. Previous reports described miR-1246 with oncogenic properties in several cancer entities (e.g. breast cancer, colon cancer, lung cancer, cervical cancer, pancreatic cancer, etc.) [36]. In all of these entities, upregulation of miR-1246 is associated with an increase in proliferation, invasion or drug resistance resulting in tumor progression [36]. In melanoma increased expression of miR-1246 was observed when compared to healthy tissue [37]. Furthermore, this study revealed that miR-1246 induces proliferation as well as migration and invasion in melanoma cells by targeting FOXA2 [37]. However, these reports focused on the intracellular expression and function of miR-1246.

Additionally, miR-1246 has been demonstrated to be enriched in EVs derived from breast cancer cells [38], lung cancer cells [39, 40], glioma cells [41], oral squamous cell carcinoma cells [42], pancreas carcinoma cells [43] and in metastatic melanoma cells [13], among others. Similar to the function of miR-1246 in cancer cells where it originated from, EV mediated delivery of miR-1246 promotes proliferation (breast cancer cells [38]), invasion (breast cancer cells [38], oral squamous cell carcinoma cells [42], glioma cells [41]) and drug resistance (breast cancer cells [38], lung cancer cells [39]). EV-bound miR-1246, originating from metastatic melanoma, has been demonstrated to enhance angiogenesis by inducing resistance to 5-fluorouracil in tumor endothelial cells [13]. In addition, circulating miR-1246 has been suggested as a biomarker for diagnosing and predicting the outcomes in pancreatic cancer [43, 44], esophageal squamous cell carcinoma [45], lung cancer [46, 47], aggressive prostate cancer [48], and melanoma [49]. In the latter, high levels of circulating miR-1246 were found to be a poor prognostic marker for response and outcome in patients with BRAF mutations receiving targeted therapy [49]. In summary, miR-1246 has been shown to be an oncogenic miRNA in various cancer types, primarily associated with migration and invasion which are major processes leading to metastasis.

To comprehend the mechanism behind the invasiveness induced by miR-1246, we wondered which mRNA might be its target. By prediction tools we identified CCNG2 as a potential target for miR-1246. We demonstrated that miR-1246 expression affects the protein levels of CCNG2 in our melanoma cell line model. Additionally, we demonstrated the direct binding of miR-1246 to the CCNG2 3’UTR by a luciferase reporter assay. Corresponding our data CCNG2 was identified as direct target of miR-1246 in oral squamous cell carcinoma [50], laryngeal squamous cell carcinoma [51], ovarian cancer [52], pancreatic carcinoma [53], colorectal cancer [54] and breast cancer [38]. In these studies, downregulation of CCNG2 by miR-1246 was associated with increased invasion and metastasis. Consistent with these findings, our analysis of publicly available data sets has shown that the expression of CCNG2 is reduced in metastatic tumors compared to primary tumors in a variety of cancer types and in particular in melanoma. Consequently, reduced expression of CCNG2 was associated with poor overall survival in pan cancers as well as in metastatic melanoma.

The linkage between miR-1246 and migration, invasion, and metastasis processes can also be associated with additional validated targets in various cancers. miR-1246 dependent downregulation of GSK-3β [40] in lung cancer cells; THBS2 [55, 56] in cervical cancer cells; CADM1 [57] and RORα [58] in hepatocellular carcinoma cells; DENND2D [42] in oral squamous cell carcinoma cells; FRK [41] in glioma cells; as well as FOX2A [37] in melanoma cells; has been shown to enhance the invasive capacity of cancer cells.

## Conclusions

In conclusion, the EV-mediated communication among tumor cells participates in the generation of new heterogeneous cell subpopulations with increased tumorigenicity. We demonstrated that transport of miR-1246 from a highly invasive subpopulation of melanoma cells contributes to an increased invasive capacity of the parental cell line. The delivery of miR-1246 results in decreased CCNG2 expression, which is known to be associated with both cancer metastasis and poor patient outcomes. Our finding provides further evidence for an oncogenic mechanism of miR-1246 in melanoma. Future research should investigate whether EV-encapsulated miR-1246 could serve as a prognostic serum biomarker or risk factor for the occurrence of metastasis in melanoma.

## Electronic supplementary material

Below is the link to the electronic supplementary material.

Supplementary Material 1

## Data Availability

The datasets analysed during the current study are available from the corresponding author on reasonable request.
