# Supplementary figures and images for: Extracellular vesicles from highly invasive melanoma subpopulations increase the invasive capacity of less invasive melanoma cells through mir-1246-mediated inhibition of CCNG2
Source: Cell Commun Signal. 2024 Sep 16;22:442. doi: 10.1186/s12964-024-01820-6 (PMC11403849; doi:10.1186/s12964-024-01820-6)

Figure 1.

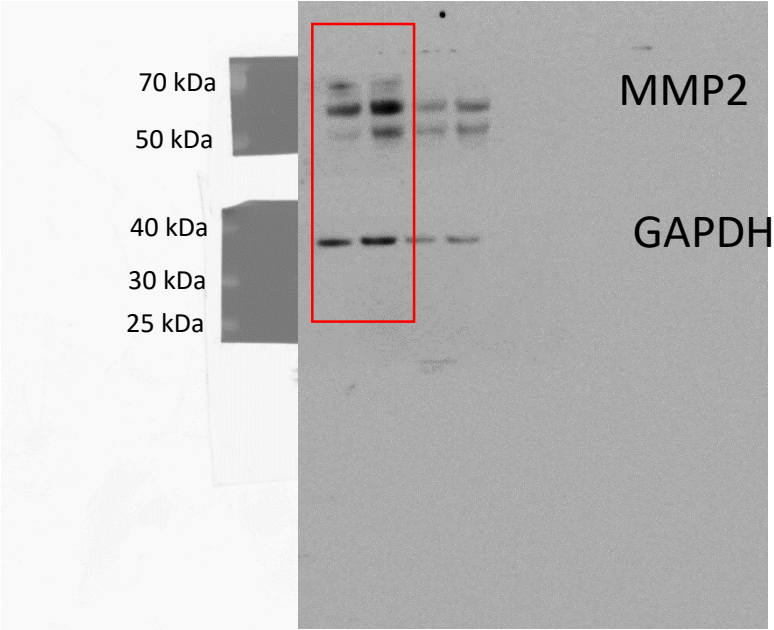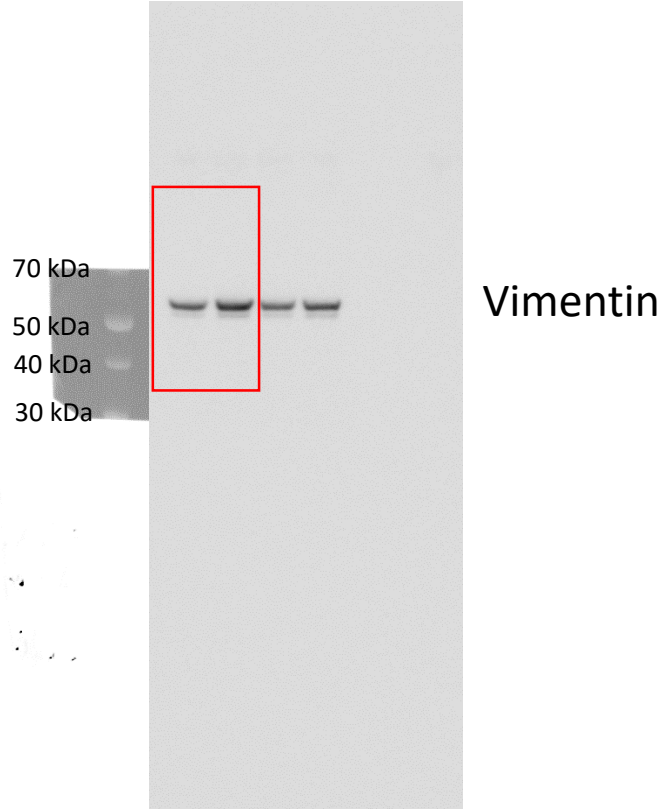

Figure 2.

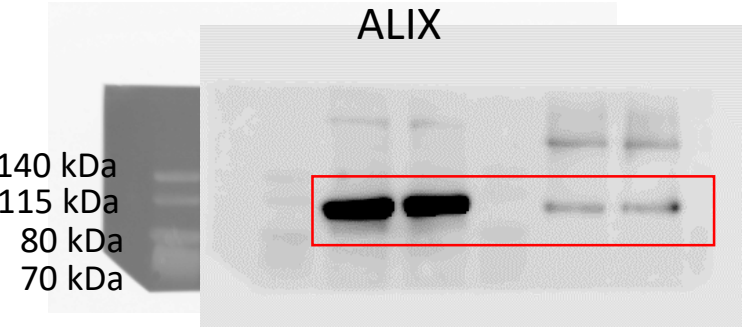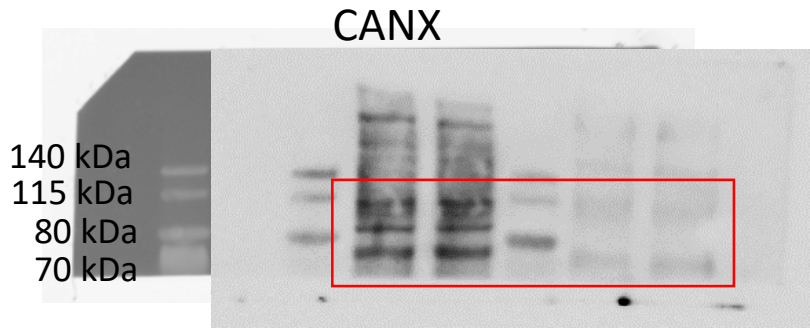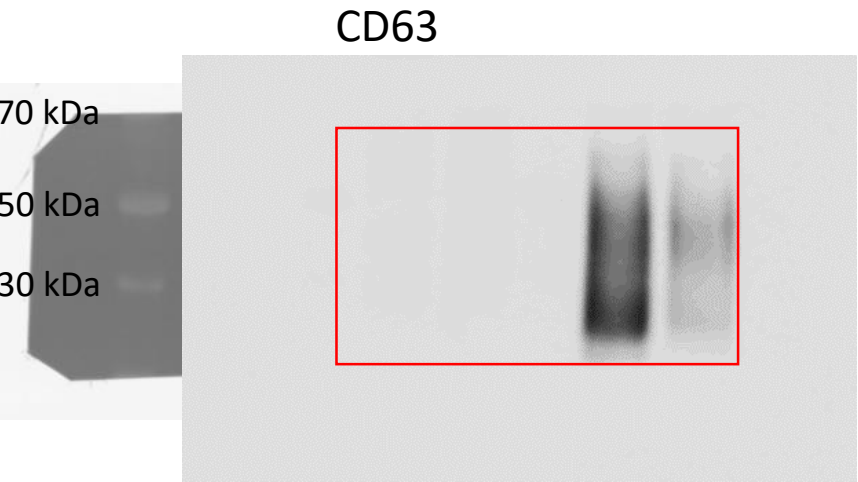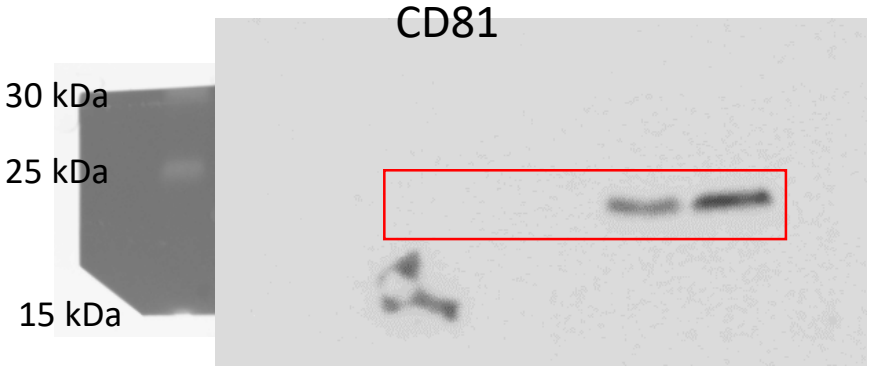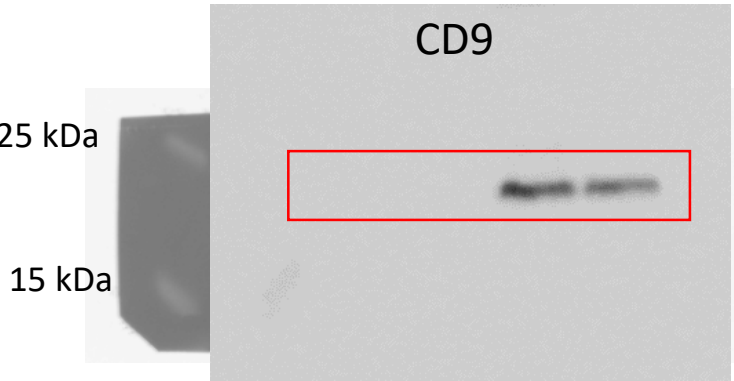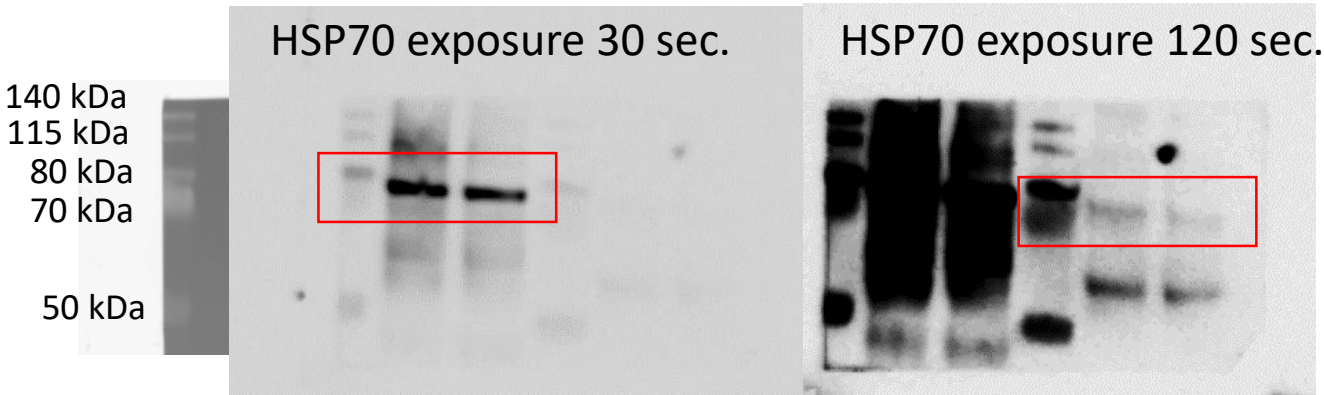

Figure 6.

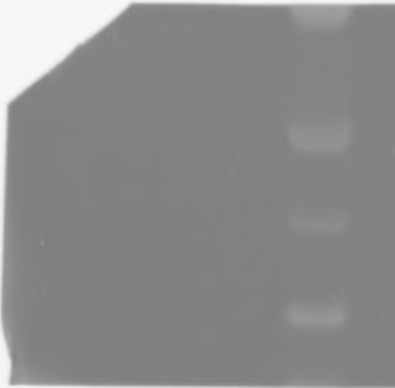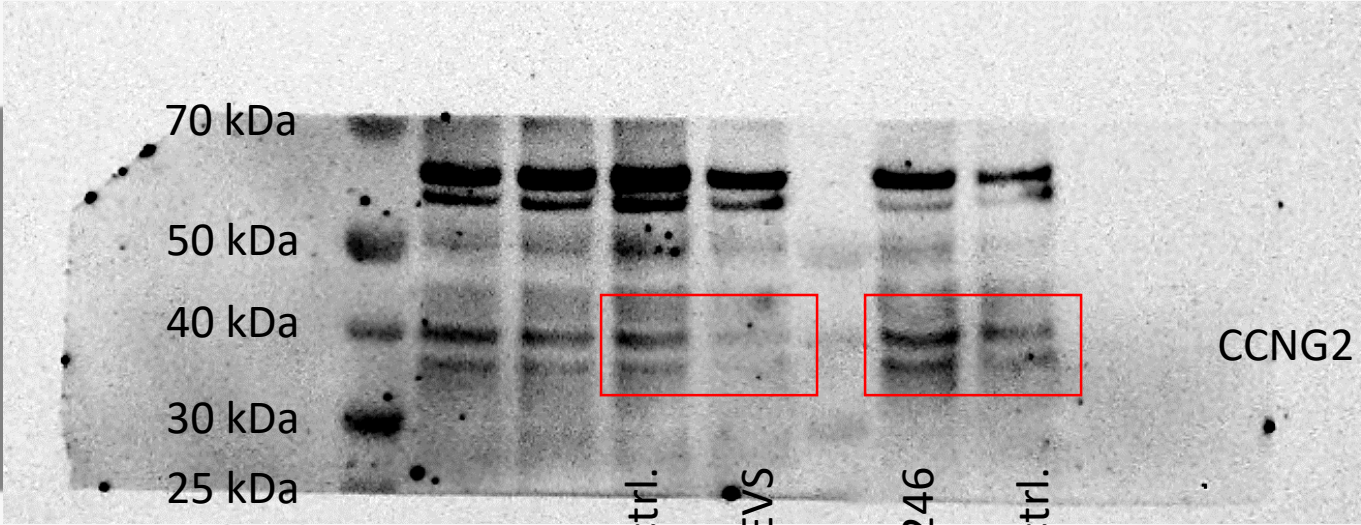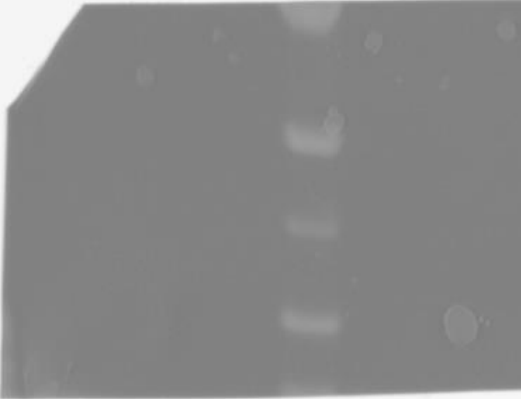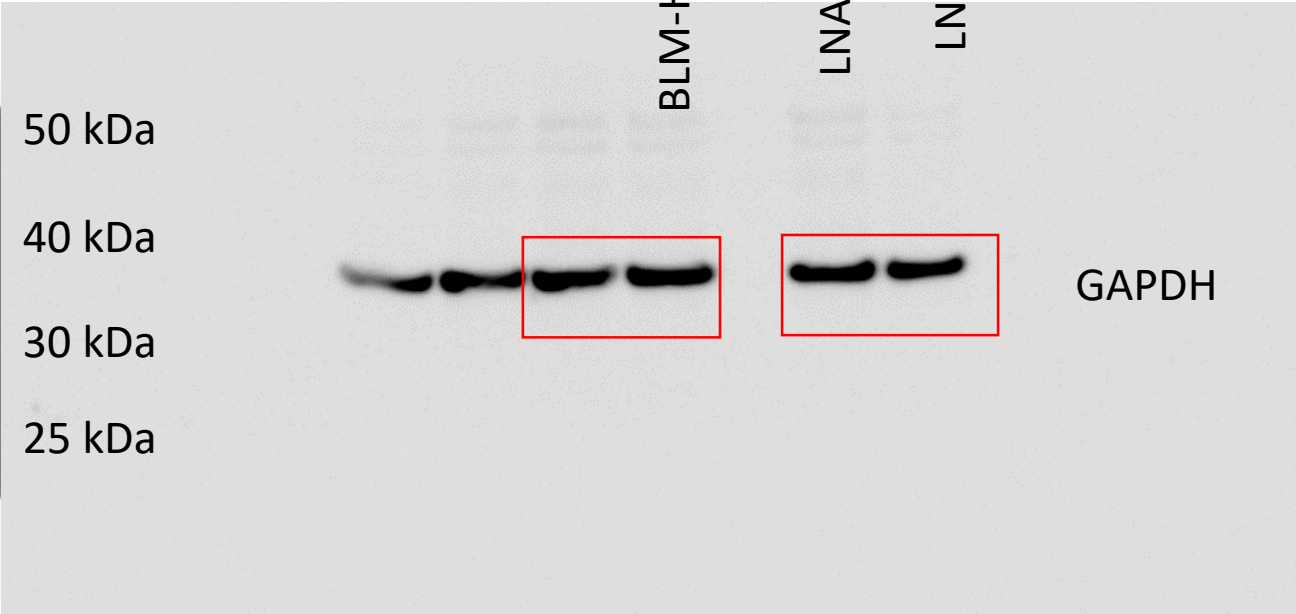

Supplement: Supplementary file 1 — Supplementary Material 1 [file 12964_2024_1820_MOESM1_ESM.pdf]
